# A culture collection of gut bacteria from wild bumble bees (*Bombus impatiens*)

**DOI:** 10.1128/mra.00791-25

**Published:** 2025-12-15

**Authors:** Kristal M. Watrous, McKenna J. Larson, Annika S. Nelson, Tobin J. Hammer

**Affiliations:** 1 Department of Ecology and Evolutionary Biology, University of California Irvine 189205, Irvine, California, USA; 2 Department of Biology, Texas Christian University 3402, Fort Worth, Texas, USA; University of Maryland School of Medicine, Baltimore, Maryland, USA

**Keywords:** symbionts, microbiome, probiotics, bumblebee, microbiota

## Abstract

Gut bacteria are important to the ecology and health of social bees. We present a collection of 55 isolates, representing a diverse range of bacterial taxa, from wild- and captive-reared bumble bees (*Bombus impatiens*). Culture conditions and full-length 16S rRNA gene sequences are provided.

## ANNOUNCEMENT

Bumble bees are an emerging model for host-microbe interactions (1, 2) and ecologically important, yet threatened pollinators (3). Much of the research in this system focuses on the “core” gut microbiome—the host-specialized bacterial symbionts that are consistently present across *Bombus* species (4, 5). Core bacteria dominate the gut microbiome of bumble bees reared indoors (6, 7). However, wild bumble bees additionally harbor a diverse and variable set of “non-core” gut bacteria that are acquired from flowers or other environmental sources (8–12). How non-core bacteria interact with bumble bees and the core microbiome is poorly understood (12). A culture collection would not only facilitate ecological research but might also aid in the development of probiotics for use in agriculture or conservation breeding programs.

We isolated 50 strains of bacteria from the gut of wild bumblebee workers (*Bombus impatiens*). They primarily belong to non-core taxa but also include five isolates of the core symbiont *Gilliamella*. Bees were collected while foraging from flowers at three sites in the midwestern and northeastern United States during the summer of 2023. Further details have been described, and the bacterial community composition of the samples (from 16S rRNA gene sequencing) has been characterized previously (12). Gut homogenates, frozen in 20% glycerol at −70°C, were spread-plated onto agar media. A few worker bee gut samples from commercial *B. impatiens* colonies (Biobest) were also plated, yielding an additional five isolates. The culture conditions for each strain are listed in Table 1. Colonies were passaged three times to obtain pure cultures and then stored as 20% glycerol stocks at −70°C.

**TABLE 1:** Taxonomy, isolation conditions, and source information for 55 bacterial isolates from the gut of *Bombus impatiens^a^*

Isolate_ID	BLAST_Genus	BLAST_species	Percent_Identity	BLAST_Hit_Accession	Isolation_Conditions	Consensus_Accession_number	SRA_Accession_number	Host	Isolation_source	Collection_date	Location
2U6	*Lonsdalea*	*quercina*	99.43%	NR_041975.1	Nutrient agar	PV951579	SRR34582332	*Bombus impatiens*	Wild bumble bee gut	18 July 2023	USA: Massachusetts, Amherst
WVU	*Hafnia*	*paralvei*	99.28%	NR_116898.1	Nutrient agar	PV951580	SRR34582337	*Bombus impatiens*	Wild bumble bee gut	18 July 2023	USA: Massachusetts, Amherst
NSH	*Hafnia*	*paralvei*	99.07%	NR_116898.1	Nutrient agar	PV951581	SRR34582336	*Bombus impatiens*	Wild bumble bee gut	18 July 2023	USA: Massachusetts, Amherst
62S	*Pantoea*	*endophytica*	99.22%	NR_178843.1	Nutrient agar	PV951582	SRR34582333	*Bombus impatiens*	Wild bumble bee gut	18 July 2023	USA: Massachusetts, Amherst
6AN	*Pseudomonas*	*parafulva*	99.93%	NR_040859.1	Pseudomonas ChromAgar	PV951583	SRR34582328	*Bombus impatiens*	Wild bumble bee gut	18 July 2023	USA: Massachusetts, Amherst
4C7	*Fructobacillus*	*fructosus*	99.93%	NR_113579.1	MRS agar with 2% fructose	PV951584	SRR34582343	*Bombus impatiens*	Wild bumble bee gut	26 August 2023	USA: Wisconsin, Madison
ZDH	*Weissella*	*cibaria*	99.72%	NR_036924.1	MRS agar with 2% fructose	PV951585	SRR34582342	*Bombus impatiens*	Wild bumble bee gut	25 August 2023	USA: Wisconsin, Madison
KF5	*Enterococcus*	*plantarum*	98.87%	NR_118050.1	Columbia blood agar, 5% CO_2_	PV951586	SRR34582361	*Bombus impatiens*	Wild bumble bee gut	25 August 2023	USA: Wisconsin, Madison
WD2	*Enterococcus*	*plantarum*	99.86%	NR_118050.1	Columbia blood agar, 5% CO_2_	PV951587	SRR34582362	*Bombus impatiens*	Wild bumble bee gut	25 August 2023	USA: Wisconsin, Madison
HRT	*Micrococcus*	*yunnanensis*	99.71%	NR_116578.1	Columbia blood agar, 5% CO_2_	PV951588	SRR34582350	*Bombus impatiens*	Captive-bred bumble bee gut	1 September 2024	USA: California, Irvine
9I3	*Staphylococcus*	*capitis*	99.92%	NR_117006.1	Columbia blood agar, 5% CO_2_	PV951589	SRR34582356	*Bombus impatiens*	Wild bumble bee gut	15 July 2023	USA: Illinois, Glencoe
MNE	*Gibbsiella*	*greigii*	99.78%	NR_126291.1	Serratia ChromAgar	PV951590	SRR34582323	*Bombus impatiens*	Wild bumble bee gut	18 July 2023	USA: Massachusetts, Amherst
3GB	*Gibbsiella*	*greigii*	99.78%	NR_126291.1	Serratia ChromAgar	PV951591	SRR34582322	*Bombus impatiens*	Wild bumble bee gut	18 July 2023	USA: Massachusetts, Amherst
NMT	*Serratia*	*ficaria*	99.42%	NR_041979.1	Serratia ChromAgar	PV951592	SRR34582325	*Bombus impatiens*	Wild bumble bee gut	26 August 2023	USA: Wisconsin, Madison
LPC	*Erwinia*	*aphidicola*	99.79%	NR_104724.1	Serratia ChromAgar	PV951593	SRR34582326	*Bombus impatiens*	Wild bumble bee gut	18 July 2023	USA: Massachusetts, Amherst
NLH	*Erwinia*	*aphidicola*	99.43%	NR_117000.1	Serratia ChromAgar	PV951594	SRR34582324	*Bombus impatiens*	Wild bumble bee gut	18 July 2023	USA: Massachusetts, Amherst
3FE	*Lonsdalea*	*quercina*	99.50%	NR_041975.1	Nutrient agar	PV951595	SRR34582334	*Bombus impatiens*	Wild bumble bee gut	18 July 2023	USA: Massachusetts, Amherst
PJF	*Lonsdalea*	*quercina*	99.50%	NR_041975.1	Nutrient agar	PV951596	SRR34582335	*Bombus impatiens*	Wild bumble bee gut	18 July 2023	USA: Massachusetts, Amherst
U8Z	*Pantoea*	*ananatis*	99.36%	NR_026045.1	Columbia blood agar, 5% CO_2_	PV951597	SRR34582369	*Bombus impatiens*	Wild bumble bee gut	18 July 2023	USA: Massachusetts, Amherst
8HR	*Gilliamella*	*bombi*	97.14%	NR_149809.1	Columbia Blood agar, 5% CO_2_	PV951598	SRR34582365	*Bombus impatiens*	Wild bumble bee gut	15 July 2023	USA: Illinois, Glencoe
JL9	*Gilliamella*	*bombi*	99.64%	NR_149809.1	Columbia blood agar, 5% CO2	PV951599	SRR34582355	*Bombus impatiens*	Wild bumble bee gut	18 July 2023	USA: Massachusetts, Amherst
L4O	*Enterococcus*	*plantarum*	99.93%	NR_118050.1	Columbia blood agar, 5% CO_2_	PV951600	SRR34582366	*Bombus impatiens*	Wild bumble bee gut	16 June 2023	USA: Wisconsin, Madison
9XK	*Pantoea*	*sp*.	92.65%	NR_041978.1	Columbia blood agar, 5% CO_2_	PV951601	SRR34582368	*Bombus impatiens*	Wild bumble bee gut	15 June 2023	USA: Wisconsin, Madison
T41	*Rosenbergiella*	*epipactidis*	99.86%	NR_126303.1	Columbia blood agar, 5% CO_2_	PV951602	SRR34582367	*Bombus impatiens*	Wild bumble bee gut	15 July 2023	USA: Illinois, Chicago Botanic Garden
UKZ	*Gibbsiella*	*greigii*	99.70%	NR_126291.1	Columbia blood agar, 5% CO_2_	PV951603	SRR34582370	*Bombus impatiens*	Wild bumble bee gut	18 July 2023	USA: Massachusetts, Amherst
Q2G	*Neisseria*	*perflava*	99.65%	NR_117694.1	Acinetobacter ChromAgar	PV951604	SRR34582371	*Bombus impatiens*	Wild bumble bee gut	16 July 2023	USA: Illinois, Chicago Botanic Garden
UBL	*Staphylococcus*	*saprophyticus*	99.86%	NR_074999.2	Glucose-calcium carbonate agar	PV951605	SRR34582344	*Bombus impatiens*	Wild bumble bee gut	16 July 2023	USA: Illinois, Chicago Botanic Garden
WYJ	*Serratia*	*nematodiphila*	99.50%	NR_044385.1	Brain heart infusion agar with 5% sheep blood	PV951606	SRR34582360	*Bombus impatiens*	Wild bumble bee gut	25 August 2023	USA: Wisconsin, Madison
Y4R	*Staphylococcus*	*succinus*	99.93%	NR_028667.1	Brain heart infusion agar with 5% sheep blood	PV951607	SRR34582349	*Bombus impatiens*	Wild bumble bee gut	25 August 2023	USA: Wisconsin, Madison
ZRR	*Gilliamella*	*bombi*	97.14%	NR_149809.1	Columbia blood agar, 5% CO_2_	PV951608	SRR34582364	*Bombus impatiens*	Wild bumble bee gut	15 July 2023	USA: Illinois, Glencoe
TPS	*Gilliamella*	*bombi*	97.09%	NR_149809.1	Columbia blood agar, 5% CO_2_	PV951609	SRR34582363	*Bombus impatiens*	Wild bumble bee gut	15 July 2023	USA: Illinois, Glencoe
JMF	*Gilliamella*	*bombi*	97.09%	NR_149809.1	Columbia blood agar, 5% CO_2_	PV951610	SRR34582359	*Bombus impatiens*	Wild bumble bee gut	25 August 2023	USA: Wisconsin, Madison
58F	*Leuconostoc*	*mesenteroides*	100.00%	NR_113251.1	MRS agar, 5% CO_2_	PV951611	SRR34582339	*Bombus impatiens*	Wild bumble bee gut	17 July 2023	USA: Massachusetts, Amherst
FUO	*Enterococcus*	*plantarum*	99.86%	NR_118050.1	MRS agar, 5% CO_2_	PV951612	SRR34582340	*Bombus impatiens*	Wild bumble bee gut	16 June 2023	USA: Wisconsin, Madison
1RG	*Arsenophonus*	*nasoniae*	98.82%	NR_042811.1	Brain heart infusion agar	PV951613	SRR34582320	*Bombus impatiens*	Wild bumble bee gut	25 August 2023	USA: Wisconsin, Madison
XWR	*Arsenophonus*	*nasoniae*	99.58%	NR_042811.1	Brain heart infusion agar	PV951614	SRR34582327	*Bombus impatiens*	Wild bumble bee gut	25 August 2023	USA: Wisconsin, Madison
B5E	*Arsenophonus*	*nasoniae*	98.82%	NR_042811.1	Brain heart infusion agar	PV951615	SRR34582318	*Bombus impatiens*	Wild bumble bee gut	25 August 2023	USA: Wisconsin, Madison
PJL	*Arsenophonus*	*nasoniae*	98.82%	NR_042811.1	Brain heart infusion agar	PV951616	SRR34582319	*Bombus impatiens*	Wild bumble bee gut	25 August 2023	USA: Wisconsin, Madison
9HC	*Lactococcus*	*lactis*	99.86%	NR_113958.1	MRS agar with 2% fructose, 5% CO_2_	PV951617	SRR34582341	*Bombus impatiens*	Wild bumble bee gut	26 August 2023	USA: Wisconsin, Madison
WQL	*Hafnia*	*paralvei*	99.58%	NR_116898.1	Enterobacteria ChromAgar	PV951618	SRR34582347	*Bombus impatiens*	Wild bumble bee gut	18 July 2023	USA: Massachusetts, Amherst
CDA	*Serratia*	*nematodiphila*	99.65%	NR_044385.1	Enterobacteria ChromAgar	PV951619	SRR34582346	*Bombus impatiens*	Wild bumble bee gut	26 August 2023	USA: Wisconsin, Madison
B24	*Pantoea*	*agglomerans*	99.89%	NR_116751.1	Enterobacteria ChromAgar	PV951620	SRR34582345	*Bombus impatiens*	Wild bumble bee gut	16 July 2023	USA: Illinois, Chicago Botanic Garden
KMI	*Apilactobacillus*	*kunkeei*	100.00%	NR_026404.1	Columbia blood agar, 5% CO_2_	PV951621	SRR34582354	*Bombus impatiens*	Captive-bred bumble bee gut	1 September 2024	USA: California, Irvine
VDQ	*Staphylococcus*	*capitis*	99.93%	NR_113348.1	Columbia blood agar, 5% CO_2_	PV951622	SRR34582357	*Bombus impatiens*	Wild bumble bee gut	15 July 2023	USA: Illinois, Glencoe
82C	*Rosenbergiella*	*epipactidis*	99.93%	NR_126303.1	Columbia blood agar, 5% CO_2_	PV951623	SRR34582358	*Bombus impatiens*	Wild bumble bee gut	18 July 2023	USA: Massachusetts, Amherst
BSJ	*Acinetobacter*	*nectaris*	99.22%	NR_118408.1	Acinetobacter ChromAgar	PV951624	SRR34582372	*Bombus impatiens*	Wild bumble bee gut	26 August 2023	USA: Wisconsin, Madison
QN2	*Lonsdalea*	*quercina*	99.23%	NR_041975.1	Columbia blood agar, 5% CO_2_	PV951625	SRR34582348	*Bombus impatiens*	Wild bumble bee gut	16 June 2023	USA: Wisconsin, Madison
C8O	*Arsenophonus*	*nasoniae*	99.64%	NR_042811.1	Brain heart infusion agar	PV951626	SRR34582321	*Bombus impatiens*	Wild bumble bee gut	25 August 2023	USA: Wisconsin, Madison
LO2	*Arsenophonus*	*nasoniae*	99.64%	NR_042811.1	Brain heart infusion agar	PV951627	SRR34582338	*Bombus impatiens*	Wild bumble bee gut	25 August 2023	USA: Wisconsin, Madison
9MN	*Staphylococcus*	*sp*.	98.28%	NR_025922.1	Pantoea genus-specific agar	PV951628	SRR34582331	*Bombus impatiens*	Wild bumble bee gut	15 July 2023	USA: Illinois, Chicago Botanic Garden
T4D	*Micrococcus*	*aloeverae*	99.42%	NR_134088.1	Pantoea genus-specific agar	PV951629	SRR34582330	*Bombus impatiens*	Wild bumble bee gut	16 July 2023	USA: Illinois, Chicago Botanic Garden
MCC	*Micrococcus*	*terreus*	98.48%	NR_116649.1	Pantoea genus-specific agar	PV951630	SRR34582329	*Bombus impatiens*	Wild bumble bee gut	25 August 2023	USA: Wisconsin, Madison
JGD.A	*Apilactobacillus*	*kunkeei*	100.00%	NR_026404.1	Columbia blood agar, 5% CO_2_	PV951631	SRR34582353	*Bombus impatiens*	Captive-bred bumble bee gut	1 September 2024	USA: California, Irvine
JGD.B	*Apilactobacillus*	*kunkeei*	100.00%	NR_026404.1	Columbia blood agar, 5% CO_2_	PV951632	SRR34582352	*Bombus impatiens*	Captive-bred bumble bee gut	1 September 2024	USA: California, Irvine
JGD.C	*Apilactobacillus*	*kunkeei*	99.93%	NR_026404.1	Columbia blood agar, 5% CO_2_	PV951633	SRR34582351	*Bombus impatiens*	Captive-bred bumble bee gut	1 September 2024	USA: California, Irvine

^*a*^ Classifications are derived from the BLAST hit with the highest percent identity value. *Pantoea* genus-specific agar is described in reference (13).

To obtain genomic DNA, 10–20 mg (wet weight) of cells were homogenized with PBS in ZR BashingBead Lysis Tubes (BeadBlaster 96, Benchmark Scientific) and extracted using a Zymo *Quick*-DNA Fungal/Bacterial DNA miniprep kit following the manufacturer’s instructions. We PCR-amplified the full-length 16S rRNA gene using primers 27F (5′-AGAGTTTGATCMTGGCTCAG) and 1492R (5′-CGGTTACCTTGTTACGACTT) with GoTaq Green Master Mix under the following conditions: denaturation at 95°C for 2 min, then 30 cycles of denaturation (95°C for 30 s), annealing (55°C for 30 s), and extension (72°C for 100 s), followed by a final elongation step at 72°C for 5 min. Amplicons were cleaned and size-selected using NucleoMag NGS magnetic beads (Takara Bio Inc., Japan). Three Sanger sequences were obtained for each isolate using the primers 27F, 1492R, and 515F (5′-GTGCCAGCMGCCGCGGTAA) on an ABI 3730xl DNA Analyzer (Azenta Genewiz). Full-length sequences were assembled using the *De Novo* Assemble tool in Geneious Prime (v.2025.0.3). The consensus sequences were trimmed using the Annotate & Predict: Trim Ends tool (error probability limit set to 0.01) in Geneious Prime, and then identified using megablast searches against the 16S ribosomal RNA (Bacteria/Archaea) database in NCBI (blast.ncbi.nlm.nih.gov, accessed 16 July 2025).

## Data Availability

16S rRNA gene consensus sequences have been deposited at NCBI (accession numbers PV951579-PV951633, see Table 1). Raw reads are available at NCBI SRA under BioProject PRJNA1293408 (accession numbers SRS25831178-SRS25831232). Isolates are freely available by email request to the corresponding author (K.M.W.) or the last author (T.J.H., hammert@uci.edu) following the completion of a Material Transfer Agreement with the University of California, Irvine.

## References

[1] Hammer TJ, Le E, Martin AN, Moran NA. 2021. The gut microbiota of bumblebees. Insect Soc 68:287–301. doi:10.1007/s00040-021-00837-1 PMC8956082 35342195

[2] Zhang Z, Zheng H. 2022. Bumblebees with the socially transmitted microbiome: a novel model organism for gut microbiota research. Insect Sci 29:958–976. doi:10.1111/1744-7917.13040 35567381

[3] Cameron SA, Sadd BM. 2020. Global trends in bumble bee health. Annu Rev Entomol 65:209–232. doi:10.1146/annurev-ento-011118-111847 31610137

[4] Koch H, Abrol DP, Li J, Schmid-Hempel P. 2013. Diversity and evolutionary patterns of bacterial gut associates of corbiculate bees. Mol Ecol 22:2028–2044. doi:10.1111/mec.12209 23347062

[5] Kwong WK, Medina LA, Koch H, Sing K-W, Soh EJY, Ascher JS, Jaffé R, Moran NA. 2017. Dynamic microbiome evolution in social bees. Sci Adv 3:e1600513. doi:10.1126/sciadv.1600513 28435856 PMC5371421

[6] Hammer TJ, Easton-Calabria A, Moran NA. 2023. Microbiome assembly and maintenance across the lifespan of bumble bee workers. Mol Ecol 32:724–740. doi:10.1111/mec.16769 36333950 PMC9871002

[7] Meeus I, Parmentier L, Billiet A, Maebe K, Van Nieuwerburgh F, Deforce D, Wäckers F, Vandamme P, Smagghe G. 2015. 16S rRNA amplicon sequencing demonstrates that indoor-reared bumblebees (Bombus terrestris) harbor a core subset of bacteria normally associated with the wild host. PLoS One 10:e0125152. doi:10.1371/journal.pone.0125152 25923917 PMC4414509

[8] Cariveau DP, Elijah Powell J, Koch H, Winfree R, Moran NA. 2014. Variation in gut microbial communities and its association with pathogen infection in wild bumble bees (Bombus). ISME J 8:2369–2379. doi:10.1038/ismej.2014.68 24763369 PMC4260702

[9] Li J, Powell JE, Guo J, Evans JD, Wu J, Williams P, Lin Q, Moran NA, Zhang Z. 2015. Two gut community enterotypes recur in diverse bumblebee species. Curr Biol 25:R652–R653. doi:10.1016/j.cub.2015.06.031 26241138

[10] Praet J, Parmentier A, Schmid-Hempel R, Meeus I, Smagghe G, Vandamme P. 2018. Large-scale cultivation of the bumblebee gut microbiota reveals an underestimated bacterial species diversity capable of pathogen inhibition. Environ Microbiol 20:214–227. doi:10.1111/1462-2920.13973 29076622

[11] Villabona N, Moran N, Hammer T, Reyes A. 2023. Conserved, yet disruption-prone, gut microbiomes in neotropical bumblebees. mSphere 8:e0013923. doi:10.1128/msphere.00139-23 37855643 PMC10732019

[12] Nelson AS, Larson MJ, Hammer TJ. 2025. Core symbionts, age at inoculation and diet affect colonization of the bumblebee gut by a common bacterial pathogen. J Anim Ecol 94:985–998. doi:10.1111/1365-2656.70029 40177853 PMC12056351

[13] Kini K, Dossa R, Dossou B, Mariko M, Koebnik R, Silué D. 2019. A semi-selective medium to isolate and identify bacteria of the genus Pantoea. J Gen Plant Pathol 85:424–427. doi:10.1007/s10327-019-00862-w

